# RalB regulates contractility-driven cancer dissemination upon TGFβ stimulation via the RhoGEF GEF-H1

**DOI:** 10.1038/srep11759

**Published:** 2015-07-08

**Authors:** Marco Biondini, Guillaume Duclos, Nathalie Meyer-Schaller, Pascal Silberzan, Jacques Camonis, Maria Carla Parrini

**Affiliations:** 1 Institut Curie, Centre de Recherche, Paris Sciences et Lettres University, 75248 Paris, France; 2 ART group, Inserm U830; 3 UMR168, CNRS, UPMC Equipe labellisée Ligue contre le Cancer; 4 Department of Biomedicine, University of Basel, Switzerland

## Abstract

RalA and RalB proteins are key mediators of oncogenic Ras signaling in human oncogenesis. Herein we investigated the mechanistic contribution of Ral proteins to invasion of lung cancer A549 cells after induction of epithelial-mesenchymal transition (EMT) with TGFβ. We show that TGFβ-induced EMT promotes dissemination of A549 cells in a 2/3D assay, independently of proteolysis, by activating the Rho/ROCK pathway which generates actomyosin-dependent contractility forces that actively remodel the extracellular matrix, as assessed by Traction Force microscopy. RalB, but not RalA, is required for matrix deformation and cell dissemination acting via the RhoGEF GEF-H1, which associates with the Exocyst complex, a major Ral effector. Indeed, uncoupling of the Exocyst subunit Sec5 from GEF-H1 impairs RhoA activation, generation of traction forces and cell dissemination. These results provide a novel molecular mechanism underlying the control of cell invasion by RalB via a cross-talk with the Rho pathway.

In the last 15 years Ral signaling pathway came to the front of the stage as a major player in human oncogenesis^1^,^2^,^3^. The small GTPase proteins RalA and RalB act downstream of the Ras oncoproteins; indeed, the direct Ral activators (RalGEFs, Guanine Exchange Factors) are direct targets of active Ras. Ral proteins have been involved in a variety of cellular processes (such as motility/invasion, apoptosis, cytokinesis and autophagy) and in various steps of tumor development (including tumor formation, survival, growth, and metastasis). Some of these functions are shared by the two Rals, others are distinct. In particular, in cancer, RalA was found to be necessary for the anchorage-independent growth of tumor cells, while RalB was shown to be critical for survival^4^, for motility^5^,^6^, and for metastasis^7^,^8^.

Concerning the role of Ral in cell migration, our laboratory showed that RalB promotes assembly of the Exocyst complex, a major Ral effector, and its localization to the leading edge of motile cells^6^. The Exocyst complex, composed of eight subunits (Sec3, Sec5, Sec6, Sec8, Sec10, Sec15, Exo70 and Exo84), mediates the targeting and tethering of post-Golgi secretory vesicles to specific membrane sites during polarized exocytosis^9^. Exocyst is molecularly connected to a Rac1 negative regulator (the RacGAP SH3BP1, GTPase Activating Protein)^10^ and to a Rac1 effector (the Wave Regulatory complex) (Biondini *et al*, manuscript in preparation). These works indicated that the RalB/Exocyst pathway contributes to spatially organize the signaling of the Rac1 GTPase, which is a master regulator of actin polymerization in lamellipodia.

For migration through an extra-cellular matrix (ECM), cells apply several invasion strategies (mesenchymal and amoeboid, single-cell and collective) which imply degradation and/or deformation of the ECM^11^. The choice among the various invasion strategies relies not only on the cell autonomous characteristics (such as genetic features), but also on the physical, cellular and biochemical properties of the tumor environment^12^,^13^. Among the many pro-invasive soluble factors present in the tumor environment, the transforming growth factor β (TGFβ) plays multi-facet roles which still await for a better comprehension in order to develop effective anti-metastasis therapies^14^,^15^. More specifically, TGFβ can promote tumorigenesis by activating the epithelial-mesenchymal transition (EMT) program^16^. EMTs physiologically take place during embryo morphogenesis allowing the conversion of stationary epithelial cells into mesenchymal cells able to migrate and invade. In the past decade, an increasing number of *ex vivo* and *in vivo* studies has provided evidences for the recapitulation of the EMT program during metastasis^17^.

In this work we aimed at understanding the interplay between two major contributors to cancer cell dissemination: the Ral pathway and the TGFβ-induced EMT. We used as cellular model the lung adenocarcinoma A549 cells that undergo a full EMT upon TGFβ treatment^18^. We found that for invasion in 2-3D Matrigel, in presence of TGFβ, A549 cells opt for a contractility-driven strategy without proteolytic degradation of the ECM, but instead with generation of traction forces on the ECM. In these conditions, we showed that the RalB/Exocyst signaling axis plays an essential role in controlling contractility by interacting with the RhoGEF GEF-H1, which stimulates the Rho-ROCK-MLC2 pathway.

## Results

### TGFβ-induced EMT promotes protease-independent dissemination of A549 cells in 2-3D

The lung adenocarcinoma A549 cells are a well-established model for *ex vivo* EMT studies. They undergo progressive EMT upon TGFβ treatment^18^,^19^. We confirmed that TGFβ treatment for 4 days induced EMT both by morphological changes (Supplementary Figure S1A) and by a switch from epithelial to mesenchymal markers (Supplementary Figure S1B). To allow the full acquisition of EMT-related invasive behaviors, we routinely treated the cells with TGFβ for at least 7 days, which allowed a complete rearrangement of actin cytoskeleton and adhesion sites to the substrate (Supplementary Figure S1C).

**Figure 1:**
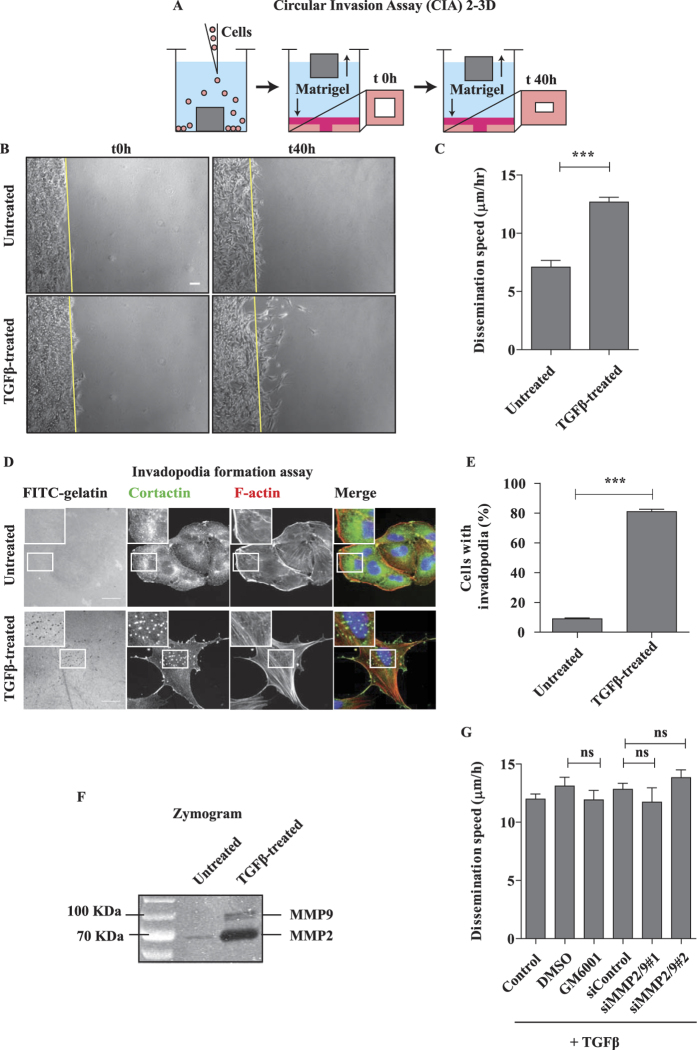
Dissemination of TGFβ-treated A549 cells in 2/3D is proteolysis independent. (**A**) Depiction of the 2/3D Circular Invasion Assay (CIA). Note that we kept the original name of the assay, but that the actual shape of the stopper we used is not circular but square. (**B**) EMT promotes cell dissemination. A549 cells were treated with 2 ng/mL TGFβ for 7 days, submitted to CIA and compared to untreated cells. Selected time points from a representative experiment are shown. See Supplementary Movie S1 for entire video sequence. Scale bar, 100 μm. (**C**) Quantification of cell dissemination. Individual cells were tracked using the ImageJ software. Number of cells n = 40 for untreated and n = 163 for TGFβ-treated conditions from at least four experiments per condition. (**D**) TGFβ greatly stimulates invadopodia formation. Cells were stimulated with TGFβ for 7 days and invadopodia were visualized as dots positive for matrix degradation (black), for cortactin staining (green) and for F-actin staining (red). Scale bar, 20 μm.(**E**) Quantification of cells positive for at least one invadopodia. Counting was performed on three independent experiments (n≥100 cells/condition per experiment). (**F**) TGFβ induces strong secretion of MMP2 and MMP9 metalloproteinases. Conditioned media from untreated and TGFβ-treated cells were collected and processed for gelatin zymogram assay. A representative zymogram image is shown. (**G**) MMP-dependent proteolysis is dispensable for TGFβ-induced dissemination in 2/3D. TGFβ-treated cells were incubated with 25 μM GM6001 MMPs inhibitor (2 hrs before CIA and during CIA) or depleted of MMP2 and MMP9, and submitted to CIA. Number of cells n>30 per condition from three experiments with GM6001 and one experiment with siRNAs. Error bars represent SEM. p values come from two-tailed Student’s t test. *p < 0.05, **p < 0.01, ***p < 0.001. ns, not significant.

To study invasion we took advantage of the Circular Invasion Assay (CIA)^20^ because it allows detailed time-lapse microscopic analysis during the invasion process (Fig. 1A). In this assay the cells are in contact with the matrix (Matrigel) both at the dorsal and frontal sides but they ventrally adhere to the bottom-glass dish; therefore it is a 2-3D situation which mimics a topography that is often observed during cancer invasion *in vivo*^21^. We compared the invasive phenotype of untreated versus TGFβ-treated cells. Untreated cells invaded through the matrix in a collective-like manner, keeping cell-cell coordination and with a rather compact front, but they were never able to invade deeply during the 2-day observation time (Fig. 1B and Supplementary Movie S1). On the contrary, TGFβ-treated cells displayed more chaotic dynamics inside the monolayer; some cells were detaching from the group and disseminating through the matrix till distant sites (Fig. 1B and Supplementary Movie S1). By single-cell tracking analysis we measured a “dissemination speed”, defined as the velocity of the cell movements in this 2-3D setting. The dissemination speed was almost 2-fold higher in TGFβ-treated cells (12.74 μm/hr ± 0.51 SEM) as compared to untreated cells (7.09 μm/hr ± 0.56 SEM) (Fig. 1C). We concluded that, in 2-3D Matrigel environment, TGFβ-induced EMT increases A549 cell invasion and promotes a shift from collective to single-cell dissemination.

The TGFβ treatment greatly stimulated invadopodia formation (Fig. 1D,E) and metalloproteinase secretion (Fig. 1F), as expected from previous reports^22^,^23^. However, very surprisingly, the dissemination speed in presence of TGFβ was not affected by the addition of a pan-inhibitor of metalloproteinases (GM6001) nor by the depletion of two major secreted metalloproteinases (MMP2 and MMP9) (Fig. 1G and S2A), indicating that TGFβ-induced invasion in 2-3D may occur via a protease-independent mechanism.

### TGFβ-treated A549 cells disseminate by a RhoA-dependent matrix deformation mechanism

A careful inspection of the videos of disseminating TGFβ-treated A549 cells in CIA revealed that they were remodeling the surrounding Matrigel matrix and leaving behind dark shadows of matrix deformation in their paths (Supplementary Movie S2). Similarly, when seeded on a thick layer of collagen gel, TGFβ-treated A549 cells clearly pulled the underlying gel in a very dynamic way, contrary to untreated cells (Supplementary Movie S3). Therefore we hypothesized that their dissemination strategy could depend on forces generated by actomyosin contractility.

**Figure 2:**
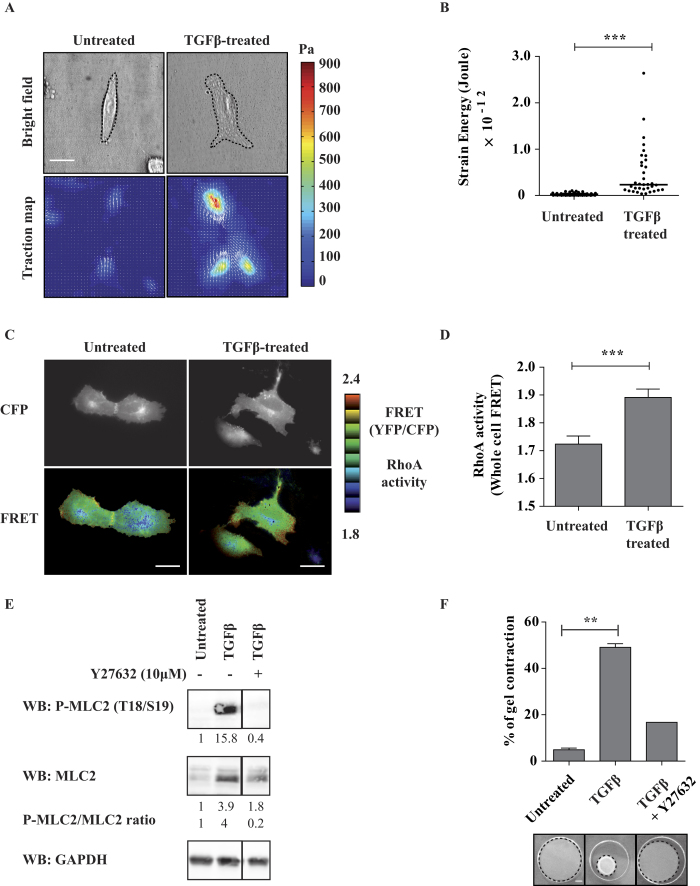
TGFβ1-induced EMT enhances actomyosin contractility and matrix remodelling. (**A**) EMT promotes traction forces on the substratum. Representative phase contrast images and corresponding traction force maps of untreated and TGFβ-treated A549 cells as revealed by Traction Force Microscopy (TFM). Color scale bar denotes traction stress (Pa). Scale bar, 50 *μ*m. (**B**) Quantification of the strain energy. TGFβ-treated cells generate significantly higher strain energies compared to untreated cells. Number of untreated cells n = 51, TGFβ-treated cells n = 35, from two experiments. (**C**) EMT stimulates Rho activity. Visualization of RhoA activity in representative untreated and TGFβ-treated cells. Cells were transfected with a plasmid expressing Raichu-RhoA (KRasCter) biosensor and observed by FRET microscopy the day after. Note the higher level of RhoA at cell periphery. Scale bars, 20 μm. (**D**) Quantification of whole-cell RhoA activity. YFP and CFP images were acquired for each cell and mean intensities were measured for the entire cell surface. The YFP/CFP ratio is a measure of whole-cell FRET, i.e. of whole-cell RhoA activity. Number of untreated cells n = 54, TGFβ-treated cells n = 41, from two experiments. (**E**) EMT induces MLC2 phosphorylation. The levels of total and phosphorylated MLC2 were analyzed by western-blot. The quantification of band intensities (with respect to untreated condition) and the calculated P-MLC/MLC ratio are indicated. The vertical lanes indicate positions were gel images were cut in order to juxtapose non-adjacent lanes coming from the same gel. (**F**) EMT stimulates ROCK-dependent actomyosin contractility. A549 cells were grown with or without TGF-β1 for 7 days and embedded in collagen I gels for 4 days. Gel contraction was monitored. Where indicated, cells were pre-treated for 2 hrs with 10 μM Y27632 ROCK inhibitor and submitted to collagen contraction assay in presence of the inhibitor. Number of untreated gels n = 2, TGFβ-treated gels n = 2, TGFβ/Y27632-treated gels n = 1, from two experiments. Error bars represent SEM. p values come from two-tailed Student’s t test. *p < 0.05, **p < 0.01, ***p < 0.001.Scale bar is 100 μm.

We took advantage of a recent biophysical technology, the Traction Force Microscopy (TFM), to provide spatially-resolved measurements of interfacial forces through the quantification of the deformation of an elastic substrate by the cells^24^. By this approach we measured the “strain energy”, which is the total energy transferred from a cell to the elastic distortion of the substrate. The strain energy of TGFβ-treated A549 cells was almost 2-fold higher than that of untreated A549 cells (0.18 pJ ± 0.02 SEM versus 0.1 pJ ± 0.01 SEM, respectively) (Fig. 2A,B). This finding indicates that TGFβ-treated A549 cells were applying much stronger traction forces on the substrate as compared to untreated A549 cells.

The RhoA pathway regulates actomyosin contractility via the ROCK kinase that phosphorylates the myosin light chain 2 (MLC2)^25^. In TGFβ-treated cells the RhoA activity was higher than in untreated cells as measured by a FRET-based biosensor (Fig. 2C,D). Consistently, the phosphorylation of MLC2 was strongly increased; noteworthy, also the level of total MLC2 was slightly increased in TGFβ-treated cells (Fig. 2E). Finally, by collagen gel contraction assays, we found that the matrix remodeling capacity of TGFβ-treated cells was much higher than that of untreated cells (Fig. 2F). As expected, the addition of the ROCK inhibitor Y27632 impaired MLC2 phosphorylation (Fig. 2E) and gel contractility (Fig. 2F).

All these data together support the conclusion that TGFβ-induced EMT increases force generation capability of A549 cells through the RhoA-ROCK-MLC2 pathway, therefore promoting dissemination in 2-3D via a contractility-dependent matrix deformation mechanism.

### RalB, but not RalA, is required for the dissemination of TGFβ-treated A549 cells

**Figure 3:**
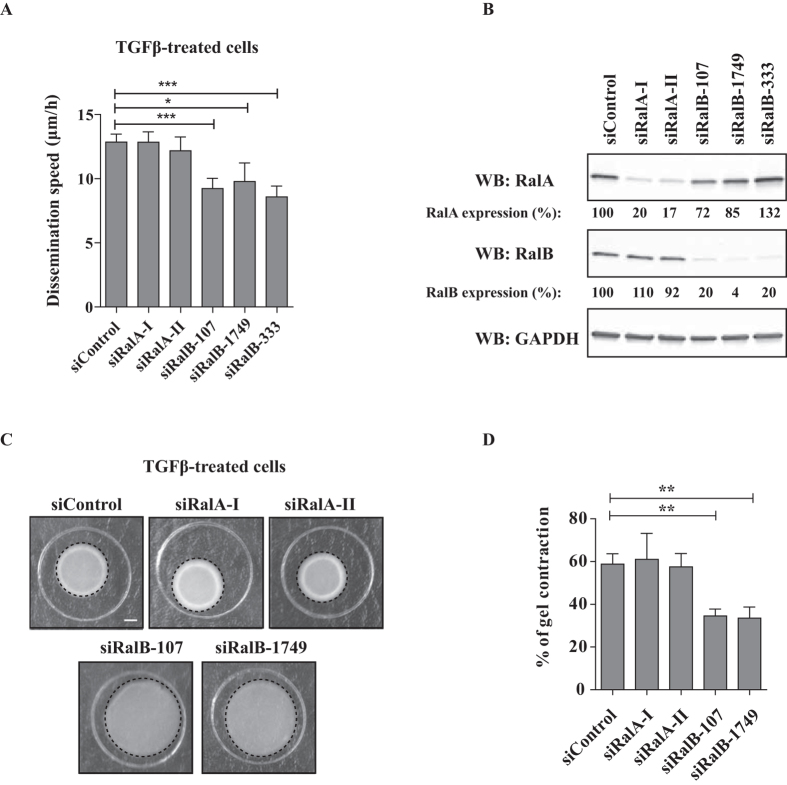
RalB regulates dissemination of TGFβ-treated cells. (**A**) RalB silencing impairs TGFβ-induced dissemination in CIA. TGFβ-treated cells were depleted of RalA or RalB, submitted to CIA and the speed of dissemination was evaluated by manual tracking. Number of cells from at least three experiments: siControl n = 76, siRalA-I n = 66, siRalA-II n = 44, siRalB-107 n = 30, siRalB-1749 n = 13, siRalB-333 n = 28. (**B**) Validation of Ral proteins depletion. Efficiency of RalA and RalB depletion was verified by western blotting.(**C**) Effect of Ral depletion on TGFβ-induced contractility. Representative images showing gel contraction by TGFβ-treated cells depleted of RalA or RalB. (**D**) Quantification of gel contraction upon Ral depletion. Mean percentage values from four to five experiments were plotted on graphic. Error bars represent SEM. p values come from two-tailed Student’s t test. *p < 0.05, **p < 0.01, ***p < 0.001.

We next investigated the role of Ral proteins in TGFβ-induced dissemination of A549 cells. TGFβ-treated cells were depleted of RalA or RalB by respectively 2 and 3 independent siRNAs and analyzed by Circular Invasion Assay (CIA). The silencing of RalB significantly impaired dissemination speed, while the silencing of RalA had no effect (Fig. 3A). The depletion levels were ≥80% (Fig. 3B). We verified that Ral depletion does not impair proliferation of TGFβ-treated A549 cells (Supplementary Figure S3A). Anyway, the strategy of tracking cells one-by-one allows us to exclude possible indirect effects of siRNA treatments on cell division and/or survival in the determination of dissemination speeds.

In addition, silencing of RalB, but not RalA, significantly impaired gel contraction capacity of TGFβ-treated cells (Fig. 3C,D). We also found that depletion of RalA or RalB does not impair secretion of MMP2 and MMP9 metalloproteinases, arguing again against a protease-dependent invasion mechanism of TGFβ-treated cells in CIA (Supplementary Figure S2B).

In conclusion, RalB but not RalA is required to regulate contractility-driven dissemination of A549 cells upon TGFβ stimulation.

### The Ral pathway mobilizes the RhoGEF GEF-H1 to drive TGFβ-induced dissemination

How RalB controls TGFβ-induced dissemination in 2-3D? Pathak and colleagues had previously shown a connection between the Ral pathway and the Rho pathway^26^. They reported that GEF-H1, a Guanine nucleotide Exchange Factor (GEF) for Rho proteins, directly binds to the Sec5 subunit of the Exocyst complex in a Ral-dependent manner. In addition, the GEF-H1/RhoA signaling activity has been linked to several processes such as cytokinesis^27^, mechano-signaling^28^,^29^, cancer motility, invasion and metastasis^30^,^31^,^32^,^33^,^34^,^35^,^36^. We reasoned that RalB could regulate TGFβ-induced dissemination by controlling Rho-driven actomyosin contractility via the Exocyst/GEF-H1 interaction.

**Figure 4:**
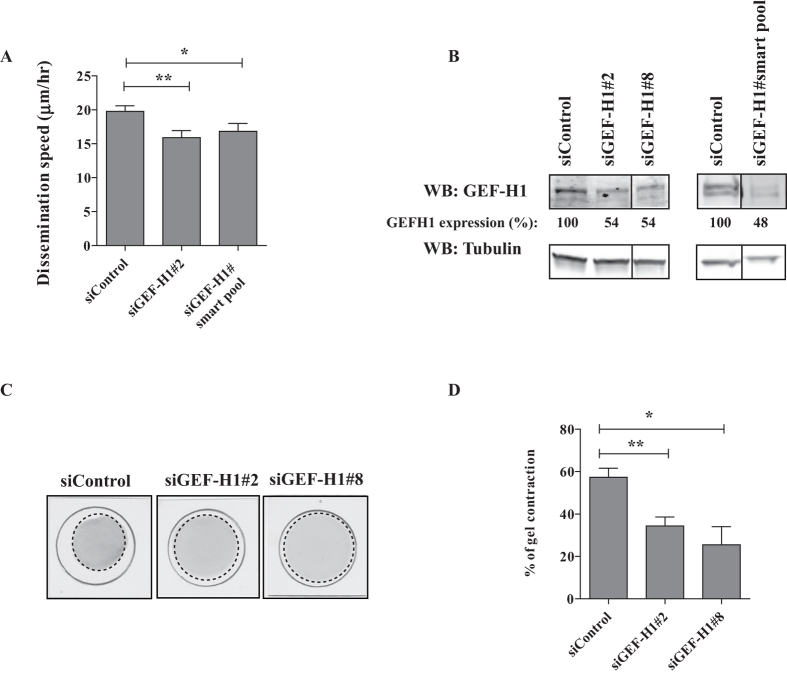
GEF-H1 controls both dissemination and contractility of TGFβ-treated cells. (**A**) GEF-H1 depletion impairs TGFβ-induced dissemination in CIA. TGFβ-treated cells were depleted of GEF-H1 and subsequently submitted to CIA. Impact of GEF-H1 silencing on dissemination speed was quantified by manual tracking. Number of cells from at least three experiments: siControl n = 81, siGEF-H1#2 n = 47, siGEF-H1#smart pool n = 32, (**B**) Validation of GEF-H1 protein depletion. Efficiency of GEF-H1 depletion was verified by western blotting. The vertical lanes indicate positions were gel images were cut in order to juxtapose non-adjacent lanes coming from the same gel. (**C**) Effect of GEF-H1 depletion on TGFβ-induced contractility. Representative images showing gel contraction by TGFβ-treated cells depleted of GEF-H1. (**D**) Quantification of gel contraction upon GEF-H1 depletion. Mean percentage values from two to six experiments were plotted on graphic.

First, we assessed the consequences of GEF-H1 depletion on dissemination and contractility of TGFβ-treated cells in our settings. Silencing of GEF-H1 by any of 2 siRNAs decreased dissemination speed (Fig. 4A) and reduced gel contraction capacity (Fig. 4C,D), despite a weak ~50% depletion efficiency (Fig. 4B). Noteworthy, GEF-H1 depletion had no significant effects on proliferation of TGFβ-treated A549 cells (Supplementary Figure S3B). These results indicate the necessity of the RhoGEF GEF-H1 for TGFβ-induced dissemination in 2-3D.

**Figure 5:**
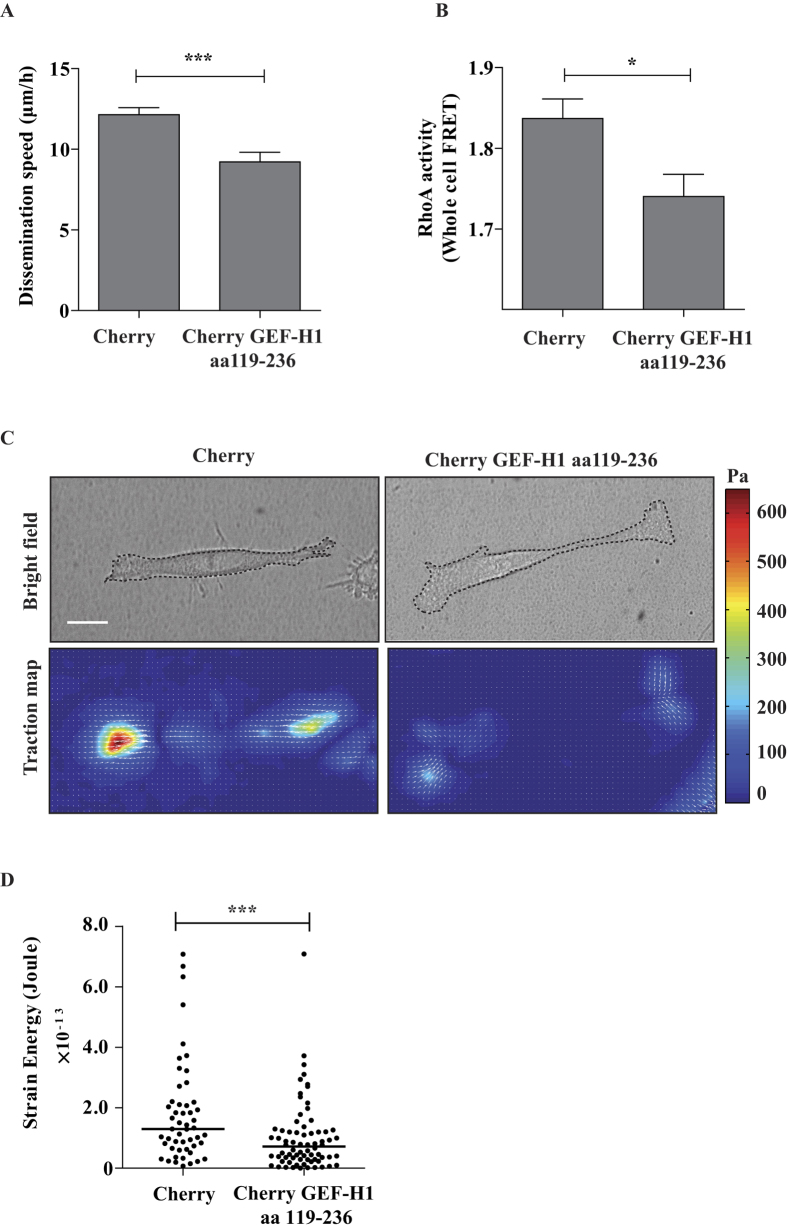
Exocyst/GEF-H1 interaction is required for dissemination, RhoA activity and generation of traction forces of TGFβ-treated cells. (**A**) Exocyst/GEF-H1 uncoupling impairs TGFβ-induced dissemination in CIA. TGFβ-treated cells stably expressing Cherry (control) or Cherry-GEF-H1^aa119–236^ (the peptide aa119–236 competes for the binding of endogenous GEF-H1 to Sec5) were submitted to CIA. Dissemination speeds were measured by manual tracking: Cherry n = 60, Cherry-GEF-H1^aa119–236^ n = 45, from two experiments. (**B**) Exocyst/GEF-H1 uncoupling perturbs RhoA activity. TGFβ-treated cells stably expressing Cherry or Cherry-GEF-H1^aa119–236^ were transfected with a FRET biosensor to visualize RhoA activity. Graphic shows mean whole-cell FRET values: Cherry n = 54, Cherry-GEF-H1^aa119–236^ n = 39, from two experiments. (**C**) Exocyst/GEF-H1 uncoupling impairs generation of traction forces. The ability of Cherry or Cherry-GEF-H1^aa119–236^ stably expressing cells to generate forces was investigated by Traction Force Microscopy (TFM). Phase contrast images and corresponding traction force maps are shown. Color scale bar denotes traction stress (Pa). Scale bar, 50 *μ*m. (**D**) Quantification of the strain energy. Cherry-GEF-H1^aa119–236^ cells generate significantly less strain energies compared to Cherry. Number of Cherry cells n = 72, Cherry-GEF-H1^aa119–236^ cells n = 51, from two experiments. Error bars represent SEM. p values come from two-tailed Student’s t test. *p < 0.05, **p < 0.01, ***p < 0.001.

Second, we perturbed the endogenous binding between the Sec5 Exocyst subunit and GEF-H1 by expressing a Cherry-fused GEF-H1^aa119–236^ competing peptide which corresponds to the minimal GEF-H1 domain interacting with Sec5^26^. Previously, this strategy was successfully used to uncouple Sec5 from GEF-H1 and to show that the Exocyst/GEF-H1 interaction modulates vesicle trafficking^26^. By lentiviral infection, we stably expressed the Cherry-fused GEF-H1^aa119–236^ competing peptide and the Cherry control in TGFβ-treated A549 cells. Comparing to the control, the expression of GEF-H1^aa119–236^ led to decreased dissemination in CIA (Fig. 5A), to decreased RhoA activity (Fig. 5B) and to decreased capacity to generate traction forces (Fig. 5C,D), without impacting on cell proliferation (Supplementary Figure S3C). These results indicate that the binding of GEF-H1 to Exocyst is necessary to activate RhoA and to promote force-driven dissemination of TGFβ-treated cells.

These data strongly suggest that RalB, via its effector Exocyst, controls contractility-driven dissemination through the RhoGEF GEF-H1, which activates the RhoA-ROCK-MLC2 pathway and promotes traction forces.

### Plasticity balance of RhoA and Rac1 pathways upon TGFβ-induced EMT

**Figure 6:**
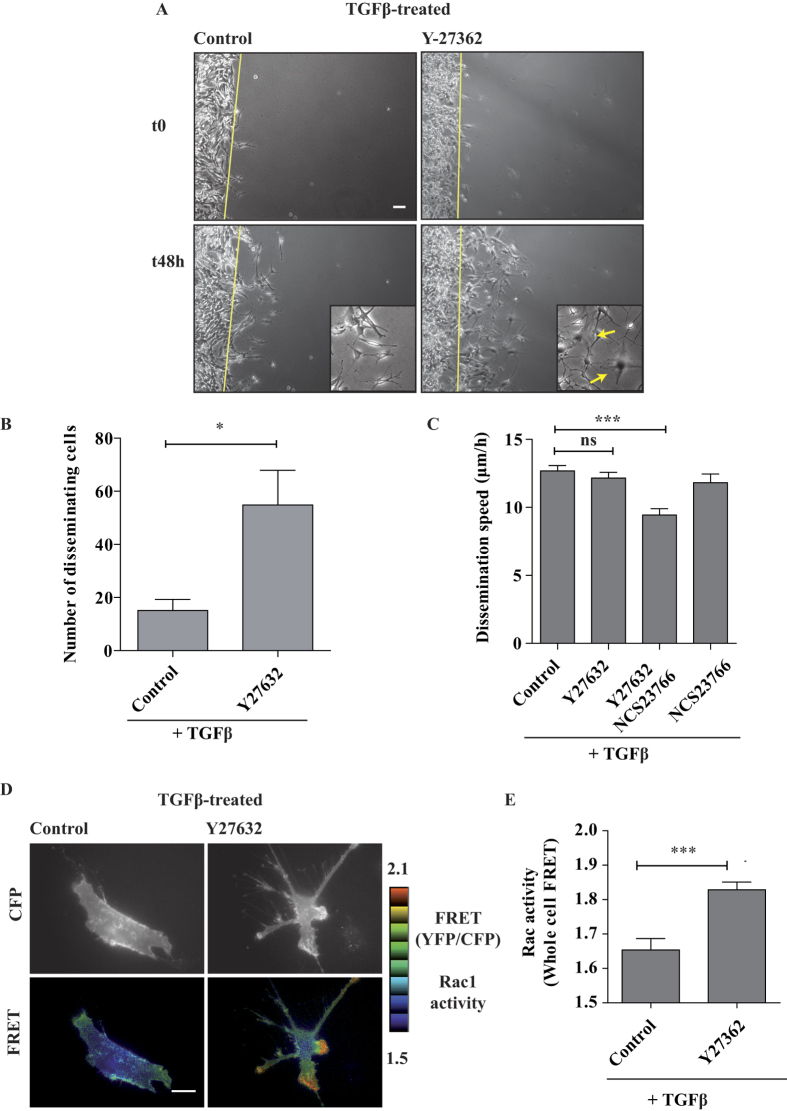
Contractility inhibition leads to Rac activation and Rac-dependent dissemination. (**A**) Inhibition of ROCK does not impair TGFβ-induced dissemination. TGFβ-treated cells were pre-treated for 2 hrs with 10 μM Y27632 ROCK inhibitor and then submitted to CIA in the presence of the inhibitor. Selected time points from a representative experiment are shown. See Supplementary Movie S3 for entire video sequence. Yellow arrows indicate broad lamellipodia-like protrusions frequently observed in TGFβ-treated cells in presence of Y27632. Scale bar, 100 μm. (**B**) Quantification of disseminating cells. The number of TGFβ-treated cells per field that in 2 days reached a distance >130 μm from the starting monolayer were counted, in absence or in presence of 10 μM Y27632, from three experiments (3 to 8 fields counted per experiment). (**C**) Rac1 activity is necessary for TGFβ-induced dissemination upon ROCK inhibition. 10 μM Y27632 ROCK inhibitor and 50 μM NCS237666 Rac1 inhibitor were added to TGFβ-treated cells as indicated. Number of cells TGFβ-treated n = 163, TGFβ/Y27632 treated n = 134, TGFβ/Y27632/NCS237666 treated n = 47, TGFβ/NCS237666 treated n = 42 from at least two experiments. (**D**) ROCK inhibition increases Rac1 activity at cellular protrusions. TGFβ-treated cells were transfected with a plasmid expressing Raichu-Rac1 (KRasCter) biosensor, treated with or without 10 μM Y27632 and visualized live by FRET microscopy the day after. Representative cells are shown. Scale bar 20 μm. (**E**) Quantification of whole-cell Rac1 activity. Number of untreated cells n = 54, Y27632 treated cells n = 33, from two experiments. Error bars represent SEM. p values come from two-tailed Student’s t test. *p < 0.05, **p < 0.01, ***p < 0.001.

The above mentioned model predicts that inhibiting the ROCK kinase would inhibit dissemination in our settings. Surprisingly, despite a strong (expected) reduction of MLC2 phosphorylation (Fig. 2E), the number of disseminating cells appeared to increase unexpectedly in presence of the ROCK inhibitor Y27632 (Fig. 6A and Supplementary Movie S4). We counted the cells that in 2 days reached a distance >130 μm from the starting monolayer and we found an increase of almost 3-fold (Fig. 6B). We obtained similar results by direct inhibition of Rho proteins with C3 transferase (Supplementary Figure S4). These results are consistent with previous work that showed that RhoA depletion promotes invasive protrusions in MDA-MB-231 cells^37^. However, the single-cell dissemination speed of TGFβ-treated A549 cells was not affected by the addition of Y27632 (Fig. 6C, control versus Y27632). Interestingly, in presence of Y27632, cells displayed broad lamellipodia-like protrusions much more frequently than in control cells, suggesting that RhoA pathway inhibition may lead to Rac1 activation as a consequence of the well-known Rho/Rac biochemical balance^37^,^38^. We tested the activation status of Rac1 in TGFβ/Y27632-treated cells by employing a FRET-based Raichu-Rac1 biosensor. We observed an up-regulation of Rac1 activity, particularly at protrusion sites (Fig. 6D). The quantifications clearly showed that the addition of Y27632 stimulated the whole-cell Rac1 activity in TGFβ-treated cells (Fig. 6E).

We reasoned that, in TGFβ-treated cells with high level of RhoA activity (Fig. 2C,D), the inhibition of Rho pathway leads to the up-regulation of Rac1 activity that may compensate the inhibition of contractility, presumably switching to a lamellipodia-driven invasion mechanism. Consistently, in TGFβ-treated cells, simultaneous inhibition of Rac1 by NSC23766 and of Rho pathway by Y27632 decreased dissemination speed (Fig. 6C, control versus Y27632/NSC23766); noteworthy, NSC23766 alone (i.e. in absence of Y27632) did not significantly impair the TGFβ-induced dissemination (Fig. 6C, control versus NSC23766), confirming that in this situation Rac1 GTPase is not at work.

These results indicate the existence of a complex balance and cross-talk between RhoA and Rac1 pathway in TGFβ-treated cells, as previously found in other cell models. In addition, these findings suggest that cancer cells, upon TGFβ-induced EMT, display a remarkable signaling plasticity which may contribute to adapt the motility/invasion programs to the chemical and physical properties of their microenvironment.

## Discussion

Tumor cells can adopt various programs to move and invade, according to their genetic background and to biochemical and physical clues from their environment. The best characterized invasion mode is linked to proteolysis: cells secrete or locally accumulate matrix metalloproteinases (MMPs) that degrade the extra-cellular matrix (ECM) and create spaces through which they can advance. This proteolysis-dependent invasion program has been widely investigated also because it is easily recapitulated *ex vivo*, for example in Transwell invasion assays. However, there is a second major mode of invasion, based on deformation of the ECM by mechanical forces, that appears to be highly relevant *in vivo*^39^. A better characterization of force-dependent invasion is recently becoming possible thanks to the development of appropriate mechanobiological approaches, such as the Traction Force Microscopy used in this study.

We found that A549 lung cancer cells after undergoing an EMT display an increased capacity to generate forces and rely on a force-driven mode of locomotion to invade ECM. This promotes cell dissemination in a 2-3D invasion assay in which cells adhere to a glass surface at the ventral side, while their dorsal and front sides face Matrigel, a gelatinous protein mixture that resembles the basement membrane. Noteworthy, this setting mimics a situation of invasion at interfaces that is not rare *in vivo*. Indeed, recent advances in intravital imaging revealed that cancer cell invasion occurs preferentially via tissue tracks of least resistance, for example along vessels, myofibers, and nerves^40^. Our results suggest that in the tumor microenvironment a local increase of TGFβ may contribute to cancer invasion and dissemination by a mechanism which relies on forces and contractility, without the need of proteolysis. We speculate that cancer cells, in particular after an EMT, may exploit this strategy to efficiently deform the pre-existing tracks and interstitial spaces surrounding tumors. The question that remains to be answered is whether the capacity to generate traction forces is a consequence of TGFβ stimulation, or of the resultant EMT, or of a combination of both. These two components cannot be easily separated.

If proteolysis of ECM is not always essential for invasion, why post-EMT cancer cells secrete great amounts of MMPs (Fig. 1F)? One possibility is that MMPs are required to do more than simply degrading physical barriers, as indicated by the emerging literature on their various regulating functions in the tumor microenvironment, including for example modulation of growth/survival, inflammatory response and angiogenesis^41^, functions that remain essential also during proteolysis-independent invasion.

**Figure 7:**
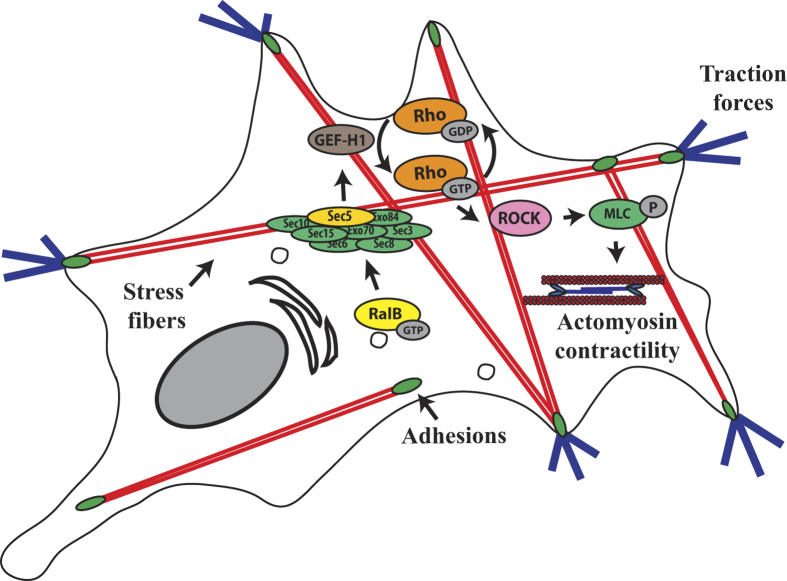
Model outlining the role of RalB/Exocyst pathway in the dissemination of TGFβ-treated A549 cells. TGFβ-treated cells generate traction forces which are required to remodel the extracellular matrix and to disseminate in 2/3D environment. This requires the activation of the Rho/ROCK pathway and actomyosin function. The Ral pathway controls the dissemination of TGFβ-treated cells by modulating RhoA-dependent actomyosin contractility via the interaction between the Sec5 subunit of Exocyst and the RhoGEF GEF-H1.

It was previously shown that RalB controls migration, invasion and metastasis. Here we showed that, in a deformation-based mode of invasion, RalB controls contractility and force generation, via the association of its effector Exocyst with the RhoGEF GEF-H1 that activates the Rho-ROCK-MLC2 pathway (Fig. 7). Among the three Rho GTPases (RhoA, RhoaB, RhoC) activated by GEF-H1^42^, we propose RhoA as the Rho player in the Ral-Rho cross-talk because the RhoA-containing biosensor monitored an increase of RhoA activating signals in TGFβ-treated A549 cells (Fig. 2C,D) and because RhoA is a well-established master regulator of cell contractility. However, a study with another cell model, the human colon LIM1863 cell line, reported a specific up-regulation of RhoC activity upon TGFβ stimulation^43^, leaving open the possibility of RhoC implication.

In clinical trials protease inhibitors failed to prove any efficacy^44^,^45^. There is therefore urgency to search for alternative strategy to block cancer invasion. Our work suggests that inhibition of the activity of RalB or GEF-H1, by decreasing contractility-driven dissemination, could have a therapeutic anti-metastatic benefit for cancer patients. On the contrary, interfering more downstream by using ROCK inhibitors may promote worse outcome, because of activation of other motility machineries (Fig. 6). Deeper investigations are needed to validate or not these predictions; in particular, concerning GEF-H1, several studies are in agreement with a pro-migratory role^30^,^31^,^32^,^33^,^34^,^35^,^36^, but others suggest an anti-invasion role^46^, depending on the motility program^33^. In addition, GEF-H1 has also been shown to be required for oncogenic MAPK signaling, independently of its RhoGEF activity^47^, further increasing the potential interest of targeting this protein in anti-cancer drug discovery. On the RalB front, very recently, small molecules inhibitors for Ral proteins have been identified^48^, which opens the path for experimentally testing in the future the effects of anti-Ral therapy *in vivo*, both in animal models and in the clinic.

Globally, these findings provide a novel mechanistic insight on how RalB contributes to force-driven cancer invasion and dissemination and suggest that RalB function may be particularly crucial after EMT. Very interestingly, choices in motility modes are not irreversible: if the Rho-based engine is perturbed, cells might opt back for Rac-based motility (Fig. 6), so strong is the cancer eagerness of movements.

## Materials and Methods

### Cell culture

A549 lung adenocarcinoma cells were cultivated in RPMI 1640 GlutaMAX (#61870-010, Invitrogen) medium supplemented with 10% fetal bovine serum (FBS), 1% penicillin/streptomycin (Invitrogen, 15070-063), 1mM sodium pyruvate (#15070-063, Invitrogen) and glucose (#G8769, Sigma, 2.8 mL in 500 mL) at 37 °C in a humidified incubator with 5% CO2. Recombinant TGFβ1 (#240-B, R&D Systems) was added to the culture medium at 2 ng/ml concentration and refreshed every 2–3 days.

### Plasmids

Raichu-RhoA 1218X and Raichu-Rac1 1011X biosensors were kindly provided by Michiyuki Matsuda (Kyoto University). To generate pLVX- Cherry-GEF-H1^aa119–236^ the sequence encoding Sec5 binding region of GEF-H1 (amino acids 119–236) was cloned as an EcoRI/BamHI fragment into pLVX lentiviral vectors (Clontech) containing mCherry (gift of Jasmine Abella).

### DNA/siRNA transfections

For DNA transfections we used JetPRIME (#POL114, Polyplus) and for siRNA transfections Lipofectamine RNAiMax (#13778030, Invitrogen) according to manufacturer’s instructions. siRNAs were purchased from Eurogentec or Dharmacon, see Supplementary Materials and Methods for sequences.

### Viral production and infection

Stable A549 cell lines expressing mCherry alone or Cherry-GEF-H1^aa119–236^ were generated via infection using lentiviruses. For viral production, 293T cells were transfected by Lipofectamine 2000 (#11668027, Invitrogen) with pLVX viral vectors along with lentiviral packaging plasmid (psPAX2) and VSVG expression vector (pCMV-VSV-G). Viral supernatant was harvested at 72 hrs post-transfection, filtered, and added to the recipient cell lines with 6 μg/ml polybrene (# 107689, Sigma-Aldrich) for 24 hrs infection. Infected cells were then selected with 2 μg/ml puromycin (# ant-pr, Invivogen).

### Circular Invasion Assay

Circular Invasion Assay (CIA) was performed as previously described^20^. Briefly, before seeding 1 × 10^6^ A549 cells a silicon self-stick cellular stopper (#80209, Ibidi) was placed in the center of a 35-mm uncoated glass bottom dish (#8158, Ibidi) to create a square space (0.80 cm^2^) devoid of cells. Upon cell adhesion, the stopper is removed and 300 μl of 50% GFR-Matrigel (#354230, Corning) (4.5 mg/ml) in 1X PBS was overlaid onto the cell monolayer seeded in the inner circle of the dish to create a matrix gel (about 0.8 mm high) which covers the dorsal part of the cells and fills the empty square space. The gel was allowed to polymerize for 2 hrs prior to addition of growth medium. Images of invading cells were acquired every 15 or 60 min using a Leica DMIR2 inverted microscope equipped with 5X or 10X objective, motorized stage, Coolsnap HQ2 camera (Roper Scientific), “Box” heated chamber and “Brick” CO2 controller (Life Imaging Services), under the control of MetaMorph software (Universal Imaging). The Manual Tracking plug-in (developed by Fabrice Cordelier) of ImageJ software was used to track cells, after which data were exported to Excel for mathematical and statistical analysis.

### FRET Imaging

Cells expressing Raichu biosensor were grown and imaged on 35-mm uncoated glass bottom dish (#8158, Ibidi). 24 hrs after transfection cells were imaged in phenol red-free MEM medium (#51200-038, Invitrogen) containing 2% FBS using a Leica DMIRE2 inverted microscope, equipped with an HCX PL APO 63× oil immersion objective, CoolSNAP HQ2 camera (Roper Scientific), filter wheels (Ludl Electronic Products), “Box” heated chamber and “Brick” CO2 controller (Life Imaging Services), under the control of MetaMorph software (Universal Imaging). For dual-emission ratio imaging, we used a D440/20× excitation filter, a 455DCLP dichroic mirror, and the two D485/40m (CFP) and D535/30m (YFP) emission filters (Chroma, Filter Set #71007a). Cells were illuminated with a 103-watt mercury lamp (Osram) through a 5% transmission neutral density (ND) filter. The exposure time was 0.05–0.2 s with a camera binning of 4 × 4. Image processing was performed using MetaMorph. For whole cell FRET measurements, the background-subtracted images were thresholded to define the whole cell surface, then the mean cell fluorescence intensities (CFP, YFP) were measured and the values were exported to Excel spreadsheets for mathematical treatment and statistics calculations. For FRET visualization, background-subtracted CFP images were thresholded to create a binary mask with a value of 0 outside the cell and a value of 1 inside the cell; after multiplication of CFP and YFP images by this mask, the FRET (YFP/CFP ratio) is represented using an eight-color scale code, as shown on the right of time-lapse figures with the upper and lower limits; the YFP/CFP ratio is used so that high ratio (red) corresponds to high Rho/Rac activity and low ratio (blue) to low Rho/Rac activity.

### Contraction assay

To assess force-mediated collagen contraction 2 × 10^5^ A549 cells were embedded in 200 μL of a collagen solution and seeded on a 35-mm glass-bottom MatTek dish (P35-1.5-14-C, MatTek). Collagen solution (1 mL) was prepared by mixing 0.8 mL of PureCol Bovine Collagen I (#5005-B, Advanced BioMatrix), 0.2 mL of 5X DMEM medium (D2429, Sigma) and 40 μL of 7.5% NaHCO3 pH = 8. After polymerization, gels with cells were maintained in culture media. Gel contraction was quantified by taking photographs (iPhone 4S) after 4 days. The relative diameters of the well and the gel were measured using ImageJ software, and the percentage of contraction was calculated using the formula: 100 x (well diameter-gel diameter)/well diameter.

### Traction Force Microscopy

Acrylamide gels containing fluorescent beads were prepared following previously published protocols^49^. The concentrations of acrylamide (polymer) and bis-acrylamide (cross-linker) were adapted to get a final Young modulus of 10.6 kPa^49^. Briefly, we placed a drop of the mixture of acrylamide/bis-acrylamide between a fibronectin-coated glass coverslip (coated with 10 mg/mL fibronectin in PBS (Fibronectin Bovine Protein, Plasma, Life technologies)) and an amino-silanated glass coverslip (silanised with 3-aminopropyltriethoxylsilane (Sigma) in isopropanol (1mL silane/50mL isopropanol)). The mixture was then crosslinked by UV light after which the top coverslip was carefully detached to obtain a gel slab attached to the silanized coverslip and coated with fibronectin. After incubating the gels for 1 hr in culture medium at 37 °C and 5% CO2, the slides were ready to be used. We seeded the cells at low density (~50 cells/mm^2^) to ensure that traction forces were measured on single cells. Gels were imaged on an inverted spinning disk confocal (Inverted Wide Confocal Spinning Disk Roper/Nikon) with a 40X oil lens. We took images of the relaxed gels after removing the cells with trypsin (Sigma). Images of the beads with and without the cells were first aligned using the ImageJ plugin “Align slices in stack”. We then used Particle Image Velocimetry (PIV) to measure the beads displacement field and the Fast Fourier Transform traction cytometry (FTTC) as previously described in^50^ in order to reconstruct the traction force field. The regularization parameter was set to 9 × 10^−10^ for all traction force reconstructions. The PIV and FFTC grid was set to 1.93 μm × 1.93 μm. Images were processed with the ImageJ software^51^. Further analysis was performed with Matlab (MathWorks, Natick, MA). To quantify cell contractility, we measured the strain energy E_strain_ which is the total energy transfered from the cell to the elastic distortion of the substrate^24^.


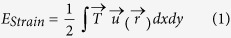



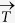
 is the traction field from the FFTC and 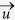 is the displacement field from the PIV measurement.

### Immunofluorescence, Immunoblotting, zymography and invadopodia assay

See Supplementary Materials and Methods.

## Additional Information

**How to cite this article**: Biondini, M. *et al.* RalB regulates contractility-driven cancer dissemination upon TGFβ stimulation via the RhoGEF GEF-H1. *Sci. Rep.* **5**, 11759; doi: 10.1038/srep11759 (2015).

## Supplementary Material

Supplementary Information

Supplementary Movie S1

Supplementary Movie S2

Supplementary Movie S3

Supplementary Movie S4
