# Supplementary material for: RalB regulates contractility-driven cancer dissemination upon TGFβ stimulation via the RhoGEF GEF-H1
Source: Sci Rep. 2015 Jul 8;5:11759. doi: 10.1038/srep11759 (PMC4495419; doi:10.1038/srep11759)
Supplement: Supplementary Information [file srep11759-s1.pdf]

RalB regulates contractility-driven cancer dissemination upon TGFβ stimulation via the RhoGEF GEF-H1

Marco Biondini[1,2], Guillaume Duclos[1,3], Nathalie Meyer-Schaller[4], Pascal Silberzan[1,3], Jacques Camonis[1,2] and Maria Carla Parrini[1,2,*]

From [1] Institut Curie, Centre de Recherche, Paris, France, [2] ART group, Inserm U830, [3] UMR168, CNRS, UPMC, [4] Department of Biomedicine, University of Basel, Switzerland

[*] Corresponding author: Maria Carla Parrini, Institut Curie, Inserm U830, 26 rue d'Ulm, 75005 Paris, France, Tel: +33156246643, email: maria-carla.parrini@curie.fr

SUPPLEMENTARY FIGURES

Figure S1

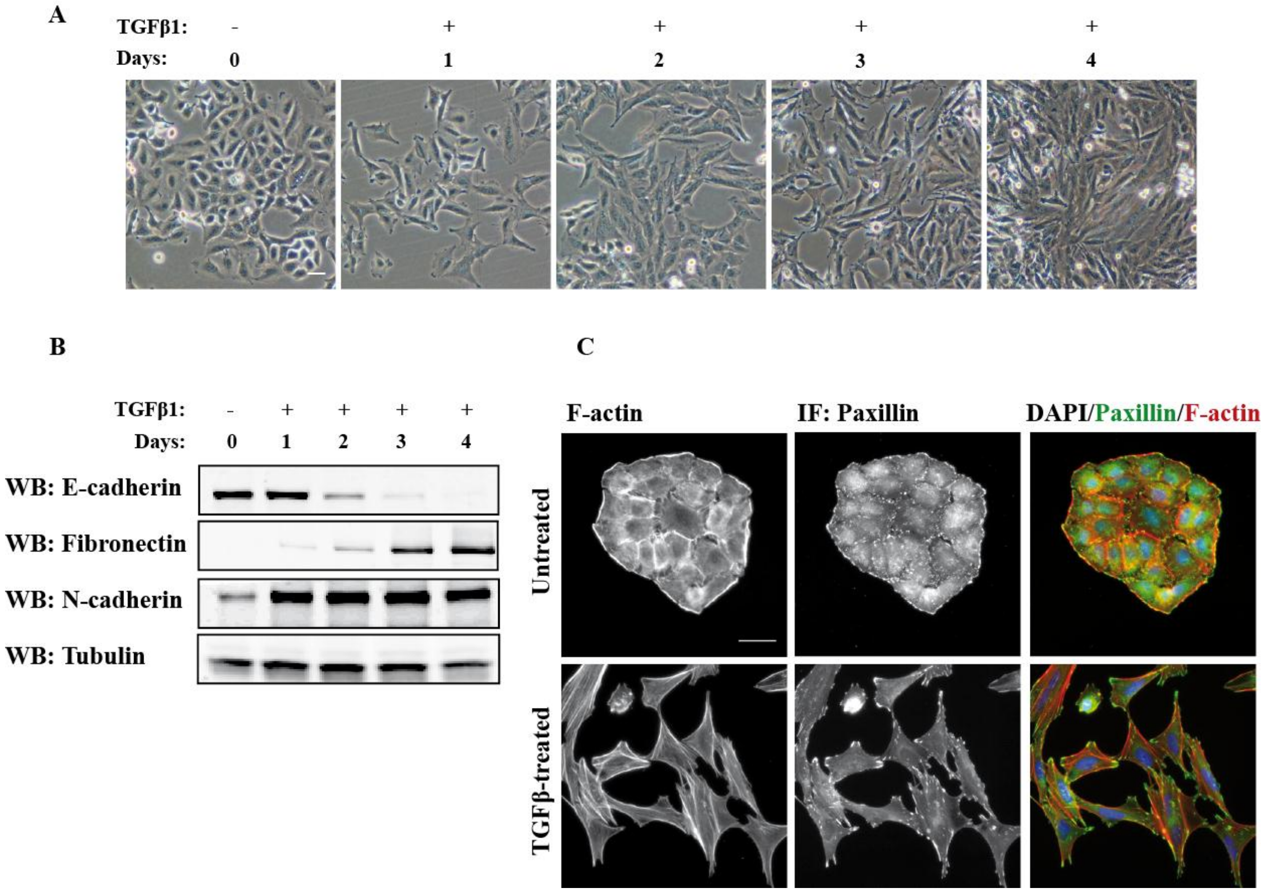

Figure S1. TGFβ induces Epithelial-Mesenchymal Transition (EMT) of A549 cells.

(A) Morphology changes. Phase-contrast images of lung adenocarcinoma A549 cells stimulated with recombinant TGFβ1 (2ng/mL) for the indicated time points. Scale bar, 20 μm.

(B) Analysis of epithelial and mesenchymal markers. Western–blot analysis of epithelial (E-cadherin) and mesenchymal markers (N-cadherin and Fibronectin) switch in A549 cells undergoing EMT.

(**C) Actin cytoskeleton and focal adhesion changes.** After 7 days of TGFβ1 treatment actin cytoskeleton was visualized by rhodamine-conjugated phalloidin (red) and focal adhesions by immunofluorescence with anti-paxillin antibody (green). Nuclei were stained with DAPI (blue). Scale bar, 20 μm.

Figure S2

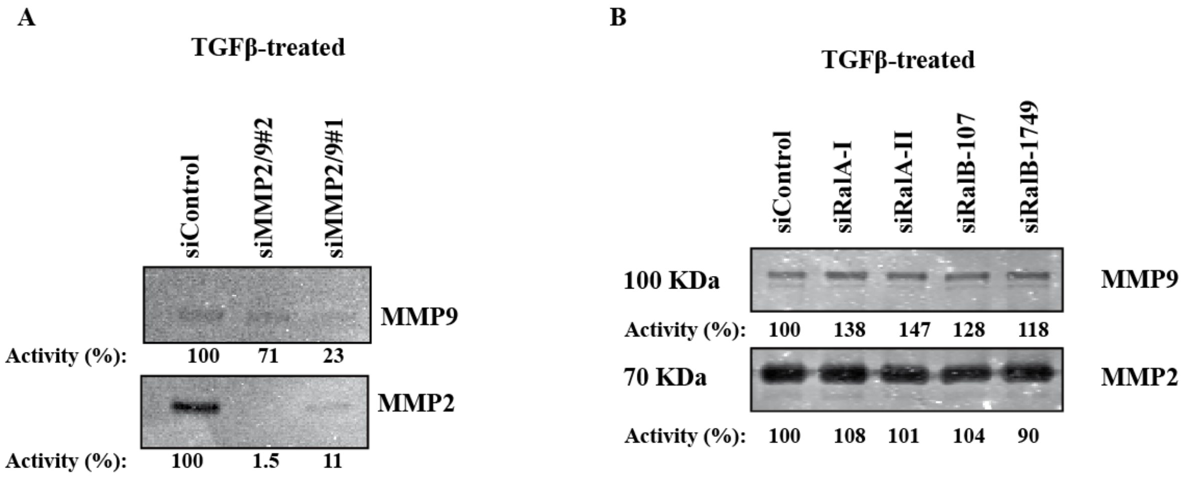

Figure S2. Supplementary zymograms.

(A) Validation of MMP2/9 depletion by siRNAs. MMP2 and MMP9 activity in conditioned medium from TGFβ-treated cells depleted of MMP2 and MMP9 was assessed by gelatin zymogram.

(B) Depletion of RalA or RalB does not impair MMP2 and MMP9 secretion. MMP2 and MMP9 activity in conditioned medium from TGFβ-treated cells depleted of RalA or RalB was assessed by gelatin zymogram.

Figure S3

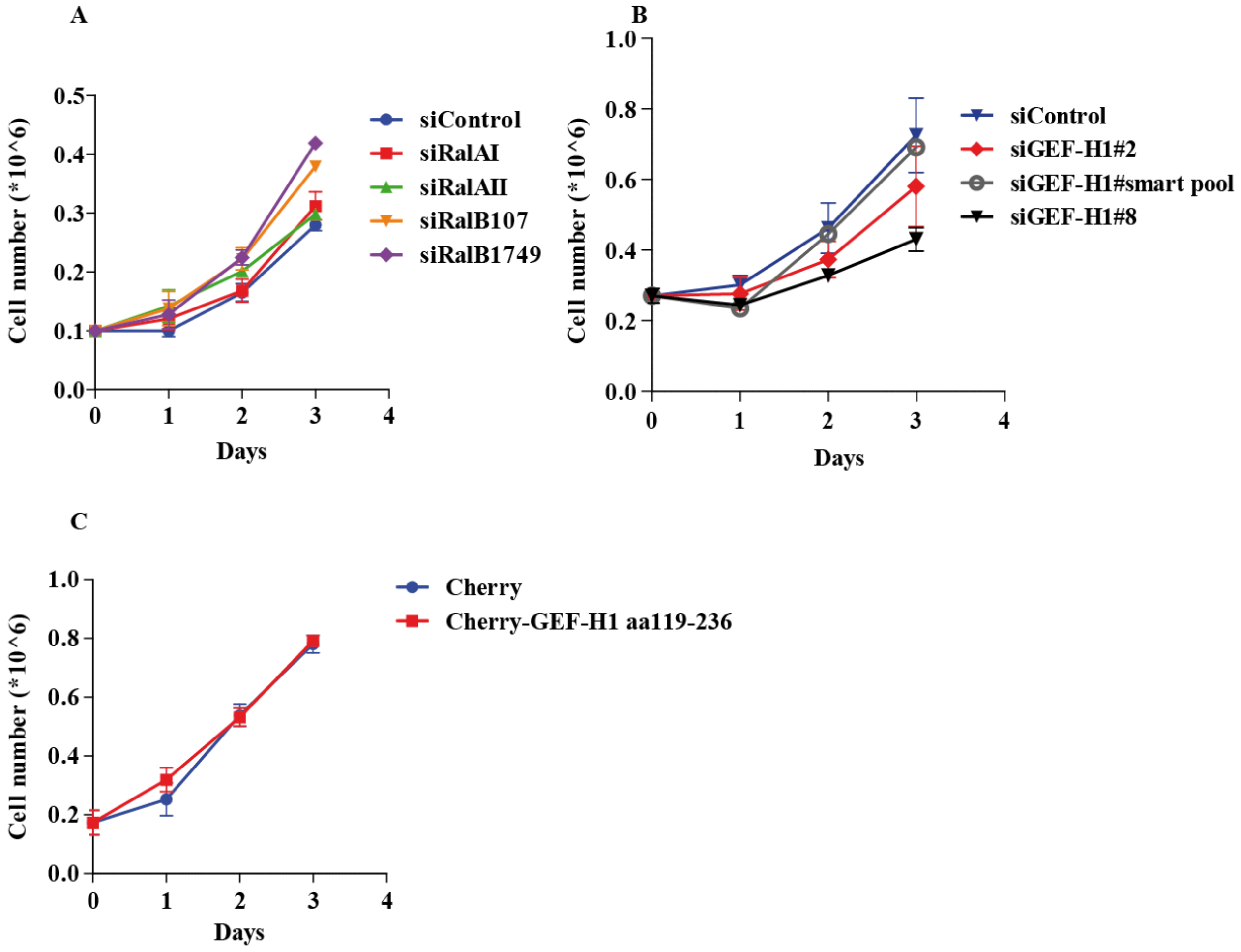

Figure S3. Impact of protein depletion or overexpression on cell proliferation.

(A) Impact of Ral depletion. TGFβ-treated cells were depleted of RalA or RalB and cell proliferation was evaluated over 3-days post-depletion by a Cell Counter.

(B) Impact of GEF-H1 depletion. TGFβ-treated cells were depleted of GEF-H1 and cell proliferation was evaluated over 3-days post-depletion by a Cell Counter.

(C) Impact of Cherry-GEF-H1$^{aa119\text{-}236}$ expression.

Figure S4

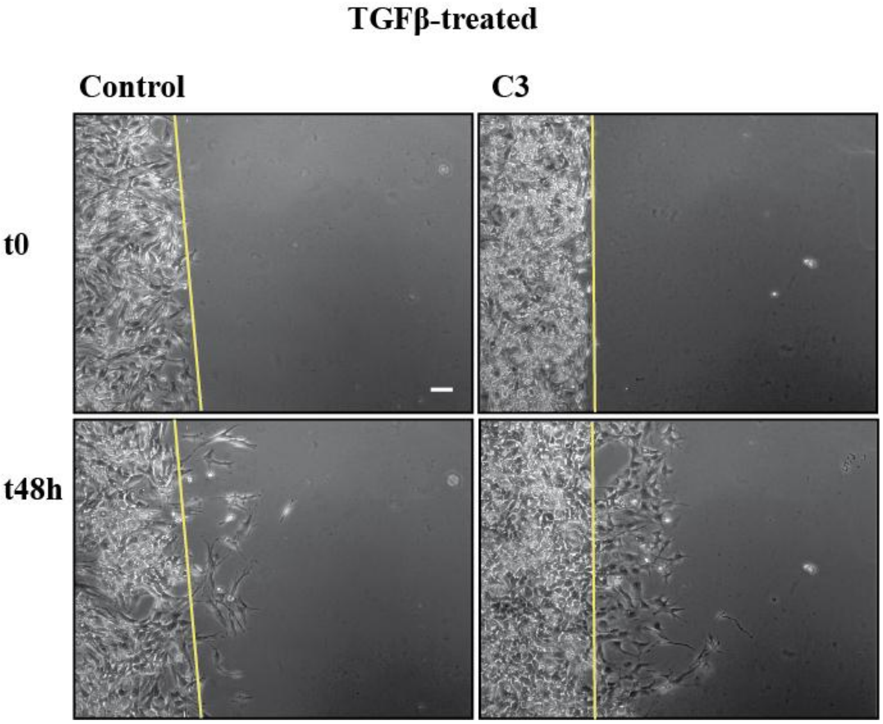

Figure S4. Inhibition of Rho proteins does not impair TGFβ-induced dissemination. TGFβ-treated cells were pre-treated for 2 hrs with 1 μg/mL C3 Rho inhibitor and then submitted to CIA in the presence of the inhibitor. Selected time points from a representative experiment are shown. Scale bar, 100 μm.

SUPPLEMENTARY VIDEOS

Movie S1. A549 disseminating in Circular Invasion Assay (CIA). Untreated (left) and TGFβ1-treated (right) A549 cells were submitted to CIA in 2-3D. Scale bar, 100 µm. Images were acquired every 15 min for 46 hrs.

Movie S2. TGFβ1-treated cells deform the matrix while disseminating in CIA.
A549 cells treated with TGFβ1 were submitted to CIA. Scale bar, 40 µm. Images were acquired every 60 min for 41 hrs.

Movie S3. TGFβ1-treated cells deform collagen matrix. Untreated (left) and TGFβ1-treated (right) A549 cells were seeded on thick collagen layers. Scale bar, 50 µm. Images were acquired every 60 min for 4 days.

Movie S4. Effect of Y27632 treatment on TGFβ1-treated cells disseminating in CIA.
TGFβ1-treated cells were treated with (right) or without (left) Y27632 and submitted to CIA. Scale bar, 100 µm. Images were acquired every 60 min for 48 hrs.

SUPPLEMENTARY MATERIAL AND METHODS

siRNA sequences

siControl, ON-TARGET plus Non-targeting siRNA #1 (#D-001810-01-50, Dharmacon)
siRalA-I, 5'-GAGACAACUACUUCCGAAGdTT-3'
siRalA-II, 5'-GACAGGUUUCUGUAGAAGAdTT-3'
siRalB-107, 5'-UGACGAGUUUGUAGAAGACdTT-3'
siRalB-1749, 5'-CAAAGACGUGAUGAGUUAATTdTT-3'
siRalB-333, 5'-CUGACAGUUAUAGAAAGAAATTdTT-3'
siGEF-H1#2, 5'-CCACGGAACUGGCAUUACUUUdTT-3'
siGEF-H1#8, 5'-GAAGGUAGCAGCCGUCUGUUUdTT-3'
siGEF-H1#smart pool, ON-TARGET plus Human ARGEF2 (# L-009883-00-0005, Dharmacon)
siMMP2/9#1:
siMMP2#A 5'-ACAAGAACCAGAUCACAUAUU-3'
siMMP9#A 5'-GCAUAAGGACGACGUGAAUUU-3'
For MMP2/9#2:
siMMP2#B 5'-GGAAUGCCAUCCCCGAUAAUU-3'
siMMP9#B 5'-GGACCAAGGAUACAGUUUGUU-3'

Immunofluorescence

Cells were fixed with 4 % paraformaldehyde for 20 min, permeabilized with 0.5% Nonidet-NP40/PBS for 5 min and blocked in 3% BSA. Primary antibodies were: mouse anti-Paxillin (#610051, Transduction labs, dilution 1:500); mouse a-Cortactin (#05-180, Millipore, dilution 1:500). Secondary antibodies were Alexa 488-conjugated anti-mouse and Cy3-conjugated anti-rabbit (Jackson ImmunoResearch). Slides were mounted with fluorescence mounting medium (#S302380, DAKO).

Immunoblotting

Cells were lysed in RIPA buffer (150 mM NaCl, 2 mM MgCl, 2 mM CaCl2, 0.5% NaDOC, 1% NP40, 0.1% SDS, 10% Glycerol, 50 mM Tris-HCL pH 8.0) containing 2 mM Na3VO4, 10 mM NaF, 1 mM DTT and a protease inhibitor mixture (#0589291001, Roche). For Phospho-myosin light chain 2 detection cells were lysed directly in 2X NuPAGE LDS sample buffer (#NP007,

Invitorgen) supplemented with 10mM NaF, 1 mM Na3VO4, 1mM DTT. Equal amounts of protein were diluted in NuPAGE LDS sample buffer and resolved by SDS-PAGE. Proteins were transferred to 0.45 µm nitrocellulose membranes (Whatman) by wet transfer and blocked with 3% BSA in TBS/0.05% Tween-20 for 30 min. Primary antibodies were: rabbit anti-Myosin Light Chain 2 (#3672, Cell Signaling, dilution 1:500); rabbit anti-phospho Myosin Light Chain 2 (Thr18/Ser19) (#3674, Cell Signaling, dilution 1:500); mouse anti-RalA (#610222, BD, dilution 1:1000); rabbit anti-RalB (#3523, Cell Signaling, dilution 1:500); mouse anti-GEF-H1(#4076, Cell Signaling, dilution 1:500). Protein levels were detected using *LICOR Odyssey* Infrared Imaging System (*LI-COR* Biosciences) upon incubation with IRDye secondary antibodies for 1hr at room temperature.

Gelatin zymography

Cells were seeded at 80% confluence and allowed to attach overnight. Serum-free medium was added to the cells for 24 hrs, conditioned media were collected. Zymogram sample buffer (#161-0764, BIORAD) was added to conditioned medium, and samples were loaded onto 10% precast polyacrylamide gels containing gelatin (#161-1167, BIORAD) and run under non reducing conditions. Protein concentrations in conditioned media were normalized to total cell numbers (total protein concentration from each cell lysate). Gels were washed with renaturation buffer for 30 min (#161-0765, BIORAD) and incubated overnight at 37°C in development buffer (#161-0766, BIORAD), stained with 0.5% Coomassie blue, 50% methanol, and 10% acetic acid, and destained in 50% methanol and 10% acetic acid. The gel was imaged using *LICOR Odyssey* Infrared Imaging System (*LI-COR* Biosciences). The presence of dark bands indicates that proteins of the corresponding molecular weights have degraded the gelatin.

In Situ Zymography and invadopodia assay

To perform in situ Zymography 18-mm coverslips were incubated with 5 µg/ml poly-L-lysine in PBS for 20 min followed by PBS washes. 0.5% gluteraldehyde/PBS was added for 10 min at room temperature, followed by PBS washes. Coverslips were inverted onto 80 µl droplets of 0.1% fluorescein isothiocyanate (FITC)-gelatin (#G13187, Invitrogen) in the dark. Coverslips were washed in PBS and then incubated 3 min in 5 mg/ml NaBH4. Coverslips were rinsed in PBS and incubated at 37°C in 10% FBS serum/DMEM for 2 hrs. 2×10^4 cells were seeded on

each coverslip, incubated for 72 hrs and processed for immunofluorescence. Invadopodia were visualized as co-localization spots of cortactin, F-actin and matrix degradation dark spots.

Cell proliferation assays

A549 cells were seeded at 2×10^5 cells/well in 12-well plates. At the indicated time points cells were trypsinized and counted by using a Vi-CELL XR (Beckman Coulter).

Culturing cells on thick collagen gels

A collagen solution at 1.7 mg/mL concentration was prepared by mixing 0.6 mL of PureCol Bovine Collagen I (#5005-B, Advanced BioMatrix), 0.2 mL of 5X medium, 7 µL of 1M NaOH and adding water to a final volume of 1 mL. Collagen solution was poured into p12 multi-well dishes (1mL/well) and let to polymerize at 37°C for 1hr. Cells were subsequently seeded at 50.000 cells/well in the appropriate culture medium.
